# Atlee’s Retrospect of Ovariotomy in Philadelphia

**Published:** 1875-03

**Authors:** 


### Bibliographical.

A RETROSPECT of the Struggles and Triumph of Ovariotomy in Philadelphia. The Annual Address before the Philadelphia County Medical Society. By Washington L. Atlee, M.D., retiring president. pp. 26.

In this address the author indulges somewhat a spirit of exultation which, we think, is not inappropriate, and may well be excused in one who has suffered so mnch, and has so signally triumphed, as Dr. Atlee has done in his self-imposed task of establishing McDowell's great operation upon a firm and sure foundation in America. We venture a few brief quotations from the address:

"It is well known that from the earliest period of ovariotomy in Philadelphia down to the present time it has been my invariable custom to invite members of the profession to witness the operation, in order that they might be able to form a proper opinion of its character, and to judge of its propriety. There was not a prominent medical gentleman in this city that had not such an opportunity. It was a rare circumstance, during the probationary stage of the operation, for any one to accept the invitation cordially and gratefully. Some did so coldly, as if conferring a favor upon me. Others politely declined. Others positively refused and emphatically condemned the operation, while others took the invitation as an insult. But after ovariotomy began to grow into favor, and since it has taken a position in legitimate surgery, an opportunity to witness it is sought after by those very individuals who were disposed to condemn it. And, what is most remarkable, the strongest opposition came from those who had never seen the operation, who would not consent to see it, and who consequently knew nothing about it; while those who reluctantly ventured to witness it, as a general rule, gradually modified their adverse opinions, and finally became advocates for it. Gentlemen, who were bold enough to witness the operation, were even directly accused by their professional acquaintances of being '*particeps criminis*' in committing murder, notwithstanding these murdered patients recovered! Some, high in the profession, against all ethical considerations, would call upon patients, who had fully decided upon the operation, for the purpose of warning them against me and certain death. * * The colleges proclaimed fiercely against the operation as unjustifiable and criminal. * * It was not uncommon for medical men to refuse to meet me in consultation, for no other reason than my persistence in in performing ovariotomy. A prominent surgeon belonging to the staff of the Pennsylvania Hospital, voluntarily said: 'Tell Dr. Atlee that I will not meet him in consultation, because he undertakes to perform operations not recognized by the profession!' Another, in passing my house, exclaimed: 'There lives the greatest quack in Philadelphia.' "

Our author alludes to a call he received from Prof. Mussey, of Cincinnati, who remarked in parting: " 'Dr. Atlee, you are yet a young man, and I would not be surprised if, during your life, you will have performed this operation *twenty* times!' This visit was very inspiring to me, and, in some measure, was a compensation for the treatment I was daily receiving from many of my professional brethren." The author may well feel, welling up from his heart, emotions of conscious pride as he contrasts the *then* and the *now* of ovariotomy, and well may we allow him to exult a little as he sums up: "The battle has been fought and won. A victory has been achieved, not alone by the prowess of the friends of ovariotomy, but because they labored for a cause which bore the seal of *truth*. No human effort can sustain an operation which has no merit in itself, and no human influence can put down an operation intrinsically good. Ovariotomy has triumphed because *truth must prevail.* The fiery ordeal through which it has passed will perpetuate the triumph of this glorious operation; and its early promoters, who have suffered trials and tribulations, may now live in peace."

